# Point-of-Care Ultrasound for Diagnosis and Pain Control of Sternal Fracture

**DOI:** 10.7759/cureus.22882

**Published:** 2022-03-06

**Authors:** Aidin Masoudi, Leily Naraghi

**Affiliations:** 1 Emergency Medicine, Maimonides Medical Center, Brooklyn, USA

**Keywords:** pain control, trauma, hematoma block, sternal fracture, point-of-care ultrasound

## Abstract

In this case report, point-of-care ultrasound (POCUS) was performed to diagnose a sternal fracture and to perform an ultrasound-guided hematoma block on an elderly patient through which excellent pain control was achieved. POCUS is a valuable tool in expediting the diagnosis of sternal fracture and can be used to safely perform a hematoma block for pain control.

## Introduction

A sternal fracture occurs due to trauma to the chest wall and can be isolated or in conjunction with other injuries [1,2]. It is reported in 8%-10% of patients suffering from blunt chest wall trauma [1-4]. These injuries can be severely painful and lead to shallow breathing, especially in elderly patients, and commonly results in the use of narcotics for pain control [2]. The reduced vital capacity and the use of narcotics can increase the risk of complications such as pneumonia and falls [5,6]. This is especially true in the elderly populations where mortality related to chest trauma, including pulmonary complications, is about 38% [5-7].

POCUS can diagnose a sternal fracture accurately and expeditiously [2,3]. Studies show that it takes less than one minute to three minutes to perform and diagnose a sternal fracture at the bedside [2,8,9]. Opioid sparing analgesia and a multimodal pain control approach are key components in preventing complications related to chest wall trauma and sternal fractures [6,10]. Ultrasound-guided sternal hematoma block is a safe and quick intervention that can play a significant role in the multimodal pain control approach [2]. This can reduce morbidity, mortality, and hospital stay for elderly patients suffering from sternal fracture [2,10].

This case report demonstrates the use of POCUS for diagnosis and pain control via hematoma block of a sternal fracture in an elderly patient.

## Case presentation

An 83-year-old female presented to the emergency department (ED) with chest wall pain after falling on a bus. She fell from standing and hit her chest on the edge of a seat. On exam, she had significant tenderness over her sternum. Her chest X-ray was unremarkable.

**Figure 1:**
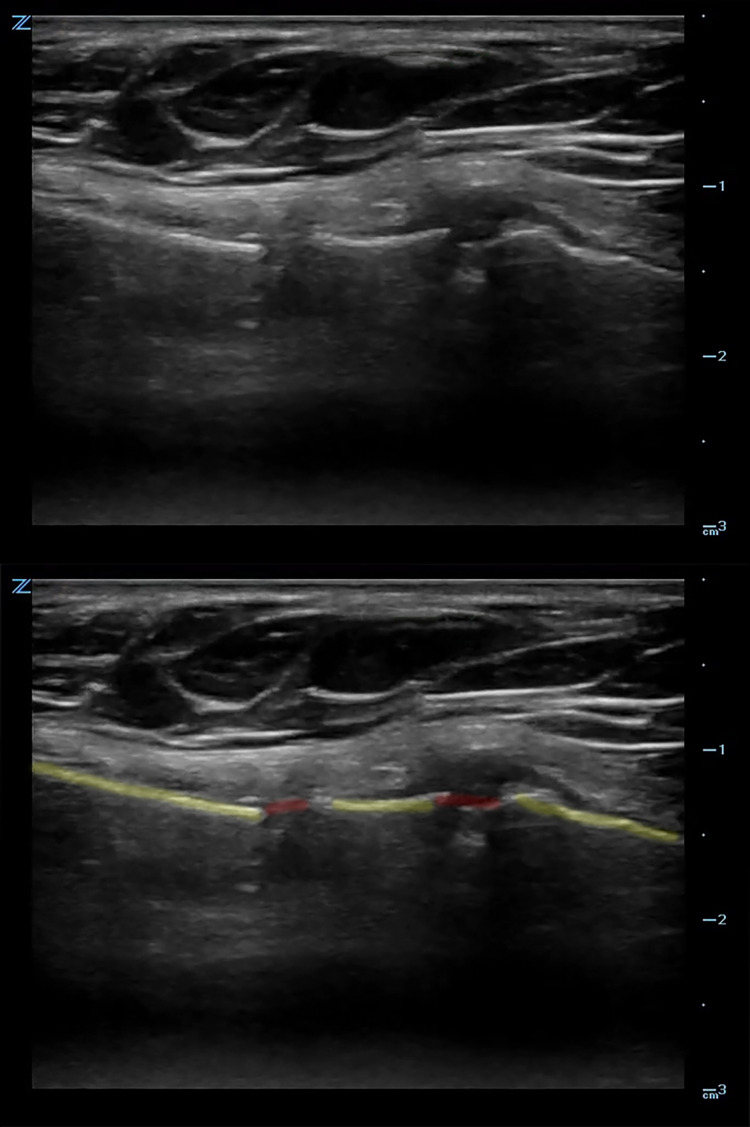
Ultrasound image of two areas of cortical disruption visualized on the sternum with overlying hematoma. The anterior cortex of the sternum (yellow) and the fracture sites (red).

Due to a strong clinical suspicion for a sternal fracture, a POCUS was performed in the ED (Video 1), revealing a sternal fracture (Figure 1).

**Video 1:** Ultrasound clip of ternal fracture.

**Figure 2:**
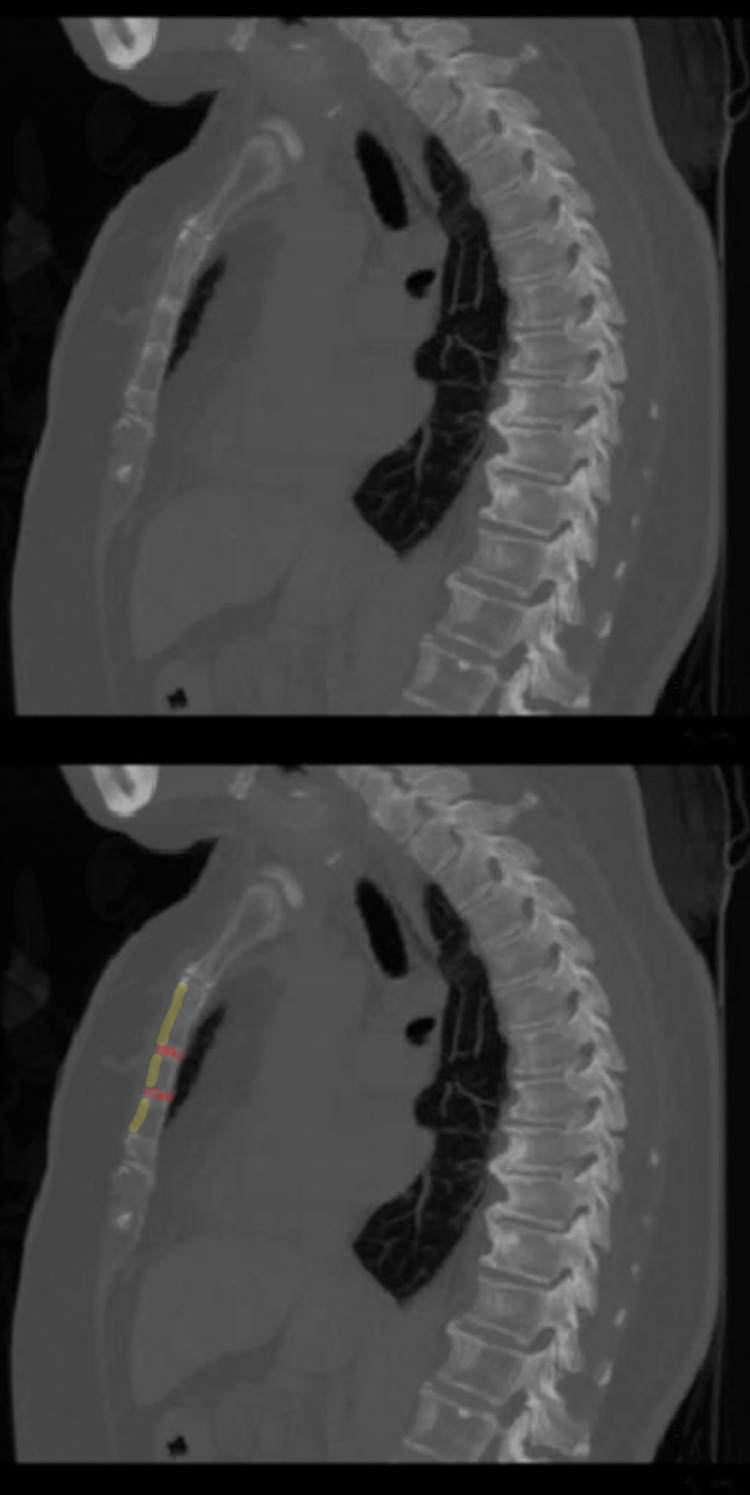
Sagittal view of the chest computed tomography. The anterior cortex of the sternum (yellow) and the fracture sites (red) are highlighted.

The sternal fracture was confirmed by chest computed tomography (CT) (Figure 2).

**Figure 3:**
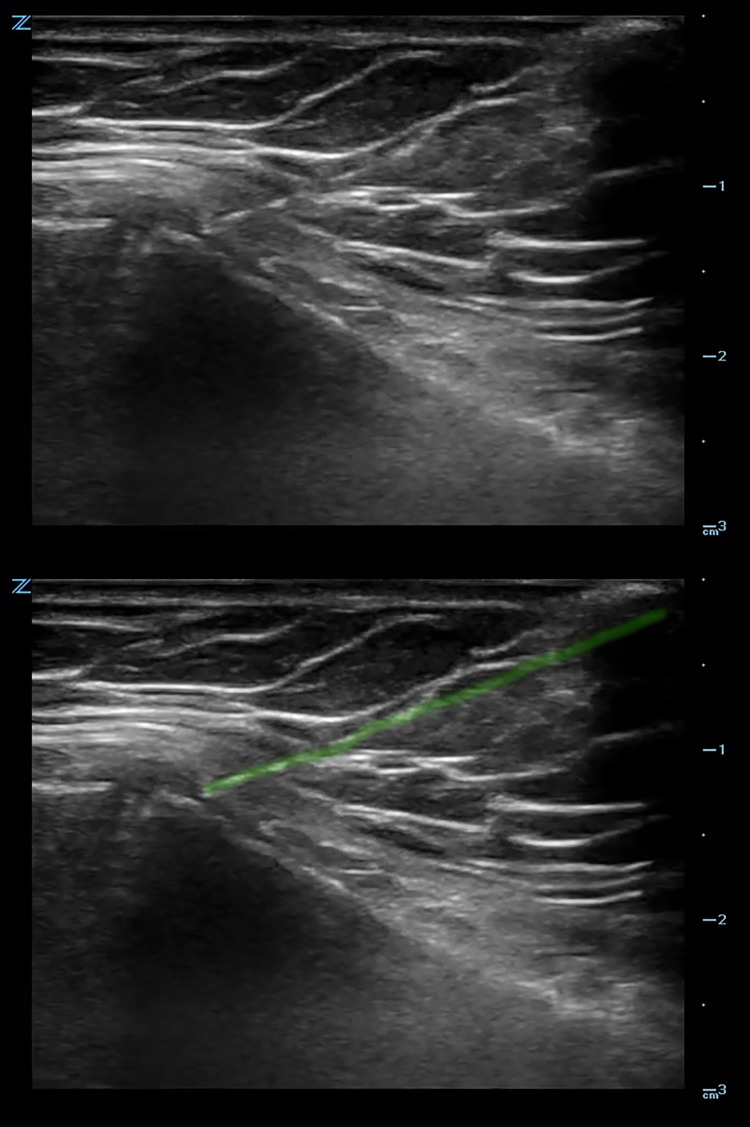
Ultrasound image of in-line needle insertion in the hematoma overlying the fracture. The needle is highlighted (green).

**Figure 4:**
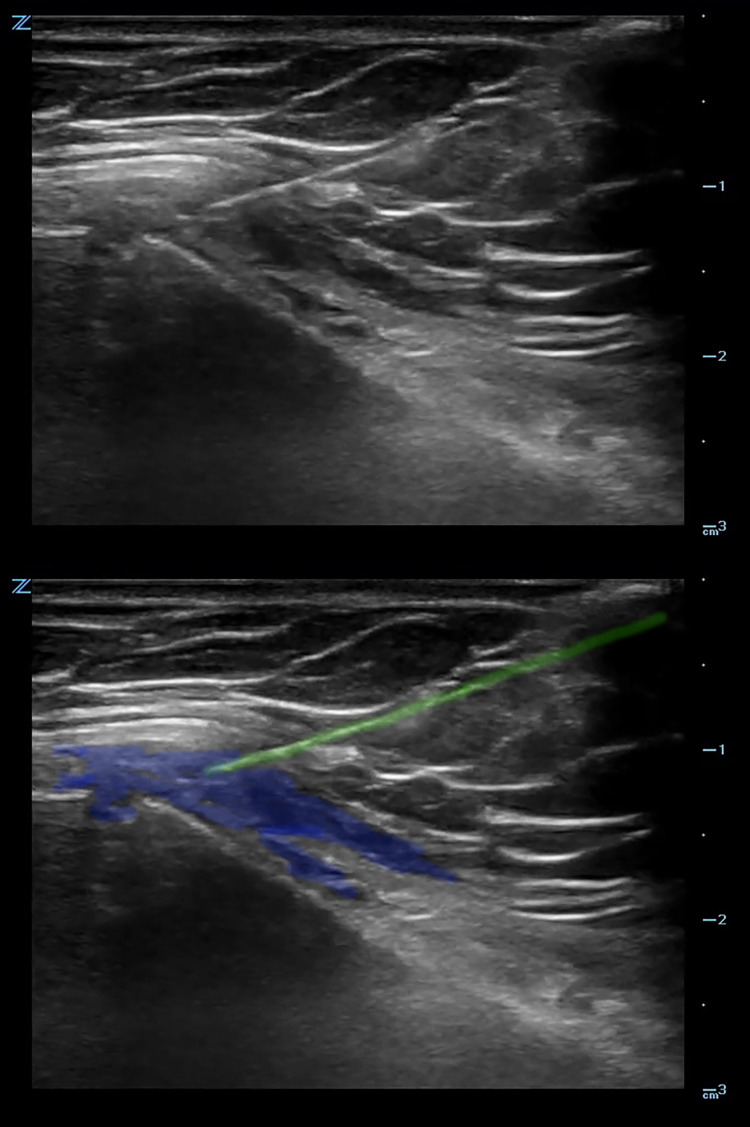
Ultrasound image after injection of local anesthetic into the hematoma. The needle (green) and the injected anesthetic (blue) are highlighted.

An ultrasound-guided sternal hematoma block was performed (Figure 3), and 10cc of bupivacaine 0.5% was injected at the site of hematoma overlying the fracture (Figure 4, Video 2).

**Video 2:** Ultrasound clip showing the needle being directed in-plane to the fracture site.

Immediately after the procedure, the patient reported complete resolution of her pain and was admitted to the hospital for 24-hour observation after which she was safely discharged home.

## Discussion

POCUS is an important tool not in the diagnosis and treatment of a variety of pathologies in the field of emergency medicine (EM). The expedited nature of POCUS, in addition to having no ionizing radiation, is extending its reach beyond EM practice. In addition, POCUS is often more accurate than X-rays for diagnosing sternal fractures, rib fractures, clavicular fractures, pneumothorax, hemothorax, and pleural effusions [2,8,9,11-15].

The flat, broad, and plane-like structure of the sternum combined with the minimal overlying soft tissue make the sternum anatomically ideal for the ultrasound diagnosis of fracture and for performing the ultrasound-guided hematoma block [2,8,9]. The limitation of ultrasound in the diagnosis of sternal fractures is the inability to estimate the severity of the injury and to rule out fracture-dislocation [2,9]. The severity of the sternal fracture can be estimated by the displacement of the fracture, which cannot be accurately measured by ultrasound when the displacement exceeds the thickness of the sternum [2,9]. This limitation makes chest CT necessary when sternal fracture-dislocation or other coexisting injuries are suspected [2].

The multimodal pain control approach is becoming a gold standard in pain management, especially in the elderly population [16]. Regional anesthesia, including ultrasound-guided hematoma block, can significantly reduce the need for opioid use in patients suffering from sternal fractures, which can reduce their mortality, morbidity, and length of hospital stay [2,6,7,10].

## Conclusions

POCUS is a valuable tool in expediting the diagnosis of sternal fractures and can be utilized to safely perform hematoma blocks for pain control.
